# Substance-dependent EEG during recovery from anesthesia and optimization of monitoring

**DOI:** 10.1007/s10877-023-01103-4

**Published:** 2023-12-18

**Authors:** Marlene Lipp, Gerhard Schneider, Matthias Kreuzer, Stefanie Pilge

**Affiliations:** Department of Anesthesiology and Intensive Care, School of Medicine, Technical University of Munich, Ismaningerstr 22, 81675 Munich, Germany

**Keywords:** Electroencephalogram, Anesthesia, Monitoring, Propofol, Inhalational anesthetics

## Abstract

The electroencephalographic (EEG) activity during anesthesia emergence contains information about the risk for a patient to experience postoperative delirium, but the EEG dynamics during emergence challenge monitoring approaches. Substance-specific emergence characteristics may additionally limit the reliability of commonly used processed EEG indices during emergence. This study aims to analyze the dynamics of different EEG indices during anesthesia emergence that was maintained with different anesthetic regimens. We used the EEG of 45 patients under general anesthesia from the emergence period. Fifteen patients per group received sevoflurane, isoflurane (+ sufentanil) or propofol (+ remifentanil) anesthesia. One channel EEG and the bispectral index (BIS A-1000) were recorded during the study. We replayed the EEG back to the Conox, Entropy Module, and the BIS Vista to evaluate and compare the index behavior. The volatile anesthetics induced significantly higher EEG frequencies, causing higher indices (AUC > 0.7) over most parts of emergence compared to propofol. The median duration of “awake” indices (i.e., > 80) before the return of responsiveness (RoR) was significantly longer for the volatile anesthetics (p < 0.001). The different indices correlated well under volatile anesthesia (rs > 0.6), with SE having the weakest correlation. For propofol, the correlation was lower (rs < 0.6). SE was significantly higher than BIS and, under propofol anesthesia, qCON. Systematic differences of EEG-based indices depend on the drugs and devices used. Thus, to avoid early awareness or anesthesia overdose using an EEG-based index during emergence, the anesthetic regimen, the monitor used, and the raw EEG trace should be considered for interpretation before making clinical decisions.

**Supplementary Information:**

The online version contains supplementary material available at 10.1007/s10877-023-01103-4.

## Introduction

Even today, anesthesia includes risks like intraoperative awareness if “too light” and adverse hemodynamic effects if “too deep” [1, 2]. Perioperative precipitants, e.g., “too deep” anesthesia with burst suppression electroencephalogram (EEG), have even been associated with an increased risk for postoperative neurocognitive disorders [3, 4]. Therefore, strategies to avoid these adverse outcomes that could come with optimizing EEG-based anesthesia monitoring are strived for. The European Society of Anaesthesiology and Intensive Care (ESAIC) guidelines on postoperative delirium and other groups recommend using a (processed) EEG to monitor the patient [5, 6]. Monitoring devices are in use to simplify the interpretation of complex EEG patterns. They translate the EEG activity into a dimensionless index (e.g., 0–100) that inversely correlates with the anesthetic level [7–9]. Each available system uses different algorithms to calculate the index. While these systems seem to function correctly during steady-state conditions, their performance may be weaker during the state transitions in and out of consciousness [10, 11]. This may, at least in parts, be based on time differences in index calculation time [11–14]. Demographic and other factors can also influence the EEG. Examples are sex [15], age [16, 17], neurological diseases [18]. Also, different anesthetic drugs induce different EEG patterns [19]. Further, a substance-dependent influence during emergence on the processed EEG has been shown before [20]. Some EEG features and their dynamics during emergence from general anesthesia are associated with the incidence of postoperative neurocognitive disorders [1] or postoperative pain [21]. However, the performance of EEG-based monitoring systems to properly track return to consciousness seems limited [10, 11]. This study was designed to analyze the index dynamics of different EEG-based monitoring systems during emergence from anesthesia that was maintained with different anesthetic regimens and to compare the systems’ indices. The systems used were the Bispectral Index (BIS, Medtronic, Dublin, Ireland), the Conox (Fresenius Kabi, Bad Homburg, Germany), and the Entropy Module (GE Healthcare, Helsinki, Finland). The patients received either balanced anesthesia with sevo- or isoflurane or total intravenous anesthesia (TIVA) with propofol.

## Methods

### Study design

For this monocentric retrospective study, we used data from a previous study [20] initiated initially to evaluate the cerebral state index (CSI) performance during the loss and return of consciousness. The Ethics Committee of the Technical University of Munich, Munich, Germany (Chairman Prof. A. Schömig) approved the study (Ethical Committee No. 1239/05). Forty-five adult patients with American Society of Anesthesiologists physical status (ASA) I or II were included between February 2005 and May 2006 after giving written consent. Following exclusion criteria were defined: history of neurological or psychiatric disease, a medication known to affect the central nervous system, including drug or alcohol abuse, the indication of a rapid sequence induction (e.g., pregnancy, emergency), planned postoperative ventilation and sedation, intolerance to any of the drugs used and difficult attachment of the EEG electrodes due to proximity to the surgical site (e.g., neurosurgical procedure, strumectomy). All patients received orthopedic surgery, including fixation of bones, joints, and ligaments or abdominal surgery at the bile ducts, the intestine, or hernias. Because of data failure, we had to exclude two patients (1x isoflurane, 1x sevoflurane) and thus analyzed the data of 43 patients.

### Clinical protocol

All patients received an induction with propofol + remifentanil/sufentanil and a neuromuscular block with atracurium or mivacurium. The attending anesthesiologist chose the maintenance regimen according to clinical standards, considering the operating procedures and the individual pre-existing conditions. The fifteen patients from each group received either propofol + remifentanil, sevoflurane + sufentanil, or isoflurane + sufentanil. Randomization was deliberately avoided to reflect standard clinical practice. During the emergence phase, most patients received metamizole (1.5–2.5 g) and some patients additionally received piritramid (3–5 mg) up to 45 min before documented ROR. All patients underwent surgery and were tested on the response to a verbal command during induction and emergence using Tunstall’s Isolated Forearm Technique [22].

### Monitoring and replay

Vitals, inspiratory oxygen, end-tidal carbon dioxide, and volatile anesthetic concentrations were measured with the Datex AS/3 (GE Healthcare, Chalfont St Giles, United Kingdom) compact monitor and stored in NeuMondD [23], together with documented events during surgery. EEG was recorded with the CSI (Danmeter A/S, Odense, DK) and the BIS A-1000. The electrodes were placed corresponding to At1, At2, FpZ (reference electrode), and Fp1 (grounding electrode) in the international ‘Ten-Twenty-System’; the impedances of the electrodes stayed below 5 k$\Omega$. The CSI and the BIS A-1000 electrodes were simultaneously placed on the patients’ forehead. So we had the raw EEG from both monitoring systems for each patient. Because the CSI has an additional high-pass recorded, we only used the BIS A-1000 EEG recordings for analysis and replay. The EEG trace, recorded with the BIS at a sample rate of 256 Hz, was replayed [24] to a BIS Vista (BIS_Vista_), a Conox QM-7000 M with the qCON index, and an Entropy Module with the state entropy (SE) as index. We collected the trend data from the BIS Vista via the live USB export option. For the CONOX, we used a mobile phone with the ConoxView app installed that was connected via Bluetooth. For the recording of the SE, we used VitalSignsCapture v1.009 software [25] and an RS-232 connection. The index recording intervals were 1 s for the BIS and Conox and 5 s for the SE.

### Emergence analysis

Emergence was defined as the period from *stop of drug supply* to *return of responsiveness* (RoR) indicated by the first repetitive motor response to a verbal command (*’Please squeeze my hand.’*). 60 s were added to RoR for the analysis to ensure that known time delays in processing [11–14] do not falsify the results. Density spectral arrays (DSA) of the emergence EEG and for a one-minute EEG episode during maintenance were plotted and compared to existing literature [19] to validate the data. To derive the power spectrum information from the EEG, we used MATLAB and the pwelch function. The DSAs were statistically compared between the regimens. The processed indices outputted by the replay were then compared between the regimens and each other regarding the course of the absolute value. Their behavior regarding clinically meaningful limits (index > 80 indicating wakefulness and index < 60 was considered an adequate surgery range) was analyzed. All indices used share the same index range of 40–60 as a recommendation for surgical intervention.

### Statistical analysis

The data was analyzed in MATLAB (Version R2021a–R2023a), and we also used the measures of effect size toolbox [26]. The following statistical analyses were performed: We used the area under the receiver operating curve (AUC) with 10k-fold bootstrapped 95% confidence intervals (CI) to analyze the performance of the median index values under the different regimens. As applied before 1, AUC > 0.7 was considered a relevant effect. A result was considered significant if the 95 % CI did not contain 0.5 [26]. Random false positives and artifact-related values were excluded from the discussion by only considering a significant difference if it occurred in a cluster of at least three time points in a row. This approach was used before [16, 27]. The cluster approach was also applied in comparing the DSAs with AUC to avoid discussing false positives. The correlation of the index values throughout emergence was evaluated with the Spearman correlation coefficient. The Kruskall-Wallis test with Tukey posthoc correction was used for the comparison of the time before RoR with an index > 80, the values at RoR, and the demographic data except for ASA status and sex, which were analyzed with a Freeman-Halton test. This manuscript adheres to the applicable STROBE guidelines.

## Results

### Patients’ characteristics

Regarding the patients’ characteristics, no significant differences between the groups regarding sex, height, and ASA status were present. The patients in the isoflurane group had a significantly higher weight than the sevoflurane (p = 0.012) and propofol (p = 0.002) group, but no significant differences in BMI. Detailed characteristics are presented in Supplemental Table S1. The patients receiving TIVA were in median 15.5–17 years older than those under isoflurane or sevoflurane anesthesia. Neither did a Fit Linear Regression Model show a dependency of [s with index > 80 before RoR] on the patients’ age (p = 0.15–0.99, R2 = 1.49e$-$5–0.158), nor did the analysis of the data of groups matched according to age bring different results than the analysis of the whole data.

### Spectral characteristics of the regimens

**Fig. 1:**
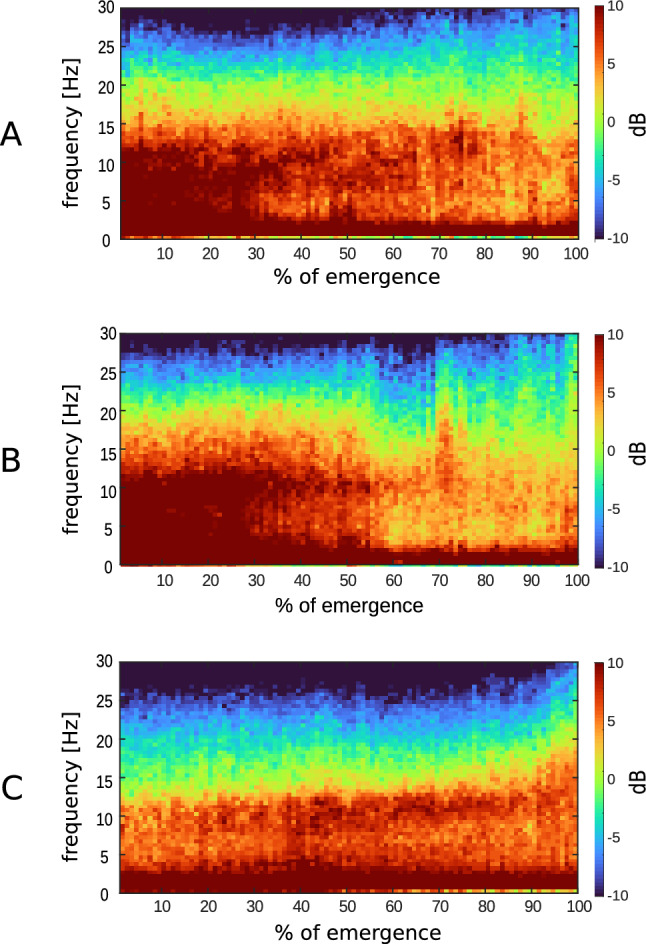
Group-level density spectral arrays (DSA) of **A** isoflurane, **B** sevoflurane, **C** propofol. Before calculating the presented group median, the emergence time of each patient was normalized to 100%

We observed significant differences between the volatiles and propofol in the EEG power spectrum during emergence as shown in the density spectral array plots (Figure 1): Sevoflurane and isoflurane-induced strong oscillatory activity in the delta (0.5–2 Hz) to alpha (8–13 Hz) band frequencies and at around 30 % of emergence the power distributes evenly over all frequencies. In contrast to the emergence from TIVA, this ‘zipper-like’ opening occurred earlier, and additionally, high power in frequencies above 20 Hz persisted throughout the emergence.

**Fig. 2:**
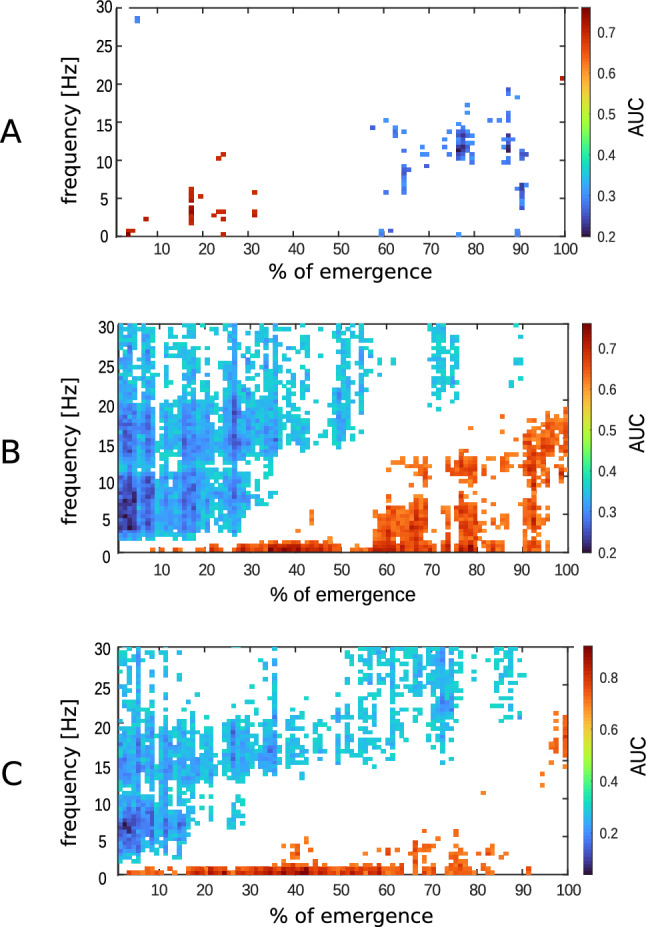
AUC for the DSA of **A** isoflurane vs. sevoflurane, **B** isoflurane vs propofol, **C** sevoflurane vs propofol. Blue pixel = significantly (*p* < 0.05) higher power in this DSA pixel of the first mentioned regimen (i.e., isoflurane in A/B, sevoflurane in C); Red pixel= significantly (p < 0.05) higher power in this DSA pixel of the second mentioned regimen (i.e., sevoflurane in A, propofol in B/C)

We applied the AUC on the DSAs of grouped data (Figure 2) and identified significantly (p < 0.05, AUC $\notin$ 0.5) higher power of the beta (14–30 Hz) and gamma (> 30 Hz) bands in the EEGs of volatile anesthetics compared to propofol during the entire time of emergence. Propofol had significantly (p < 0.05, AUC $\notin$ 0.5) higher power in the slow delta band until immediately before RoR.

### The behavior of the processed EEG indices

**Fig. 3:**
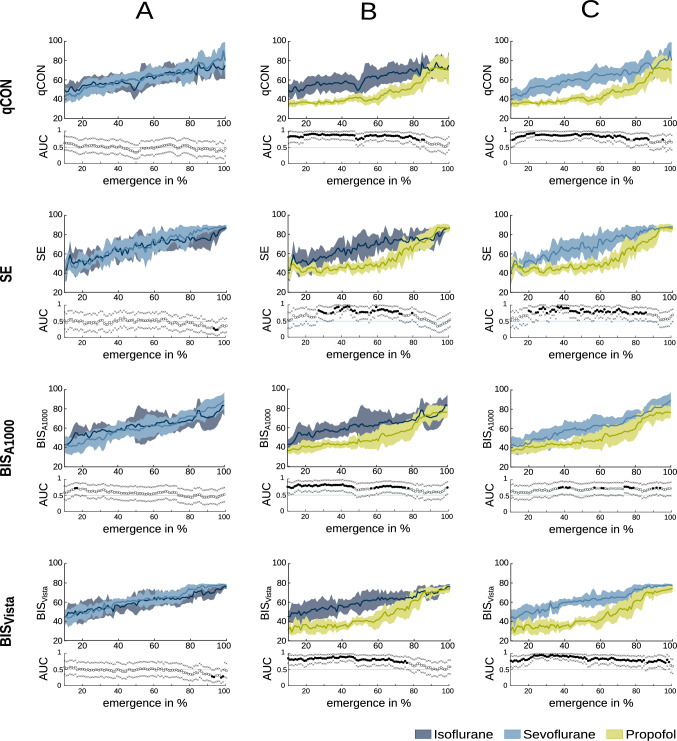
Index values of all patients per regiment group **A** isoflurane vs sevoflurane, **B** isoflurane vs propofol, **C** sevoflurane vs propofol. Thick line: median; AUC plots: a filled circle in black indicates significance, and a gray circle indicates a non-significant AUC > 0.7. The non-filled circles indicate AUC < 0.7 with 95 % CI inclusive 0.5, i.e., there is no effect. The x indicates the upper and lower limits of the 95 % CI

The indices all showed a similar trend behavior with rising values towards RoR. Still, there were significant differences between the regimens, as verified by AUC (Figure 3): Relevant and significantly (p < 0.05, AUC $\notin$ 0.5) higher values of volatile anesthetics than TIVA were present for the percentages of emergence listed in (Table 1).

**Table 1:** Indices depending on anaesthetic regimen

	Iso > Prop (%)	Sevo > Prop (%)	Iso </> Sevo (%)
BIS_A1000_	57	21	2
BIS_Vista_	65	87	4
SE	44	59	3
qCon	75	76	0

Percentage of emergence in which isoflurane (Iso > Prop) and sevoflurane (Sevo > Prop) induce clearly higher index values (AUC > 0.7, CI $\notin$ 0.5) than propofol on the corresponding monitor devices. The analyzed period is 10–100 % of emergence, as the first 10 % of emergence was cut off due to replaying artifacts

**Fig. 4:**
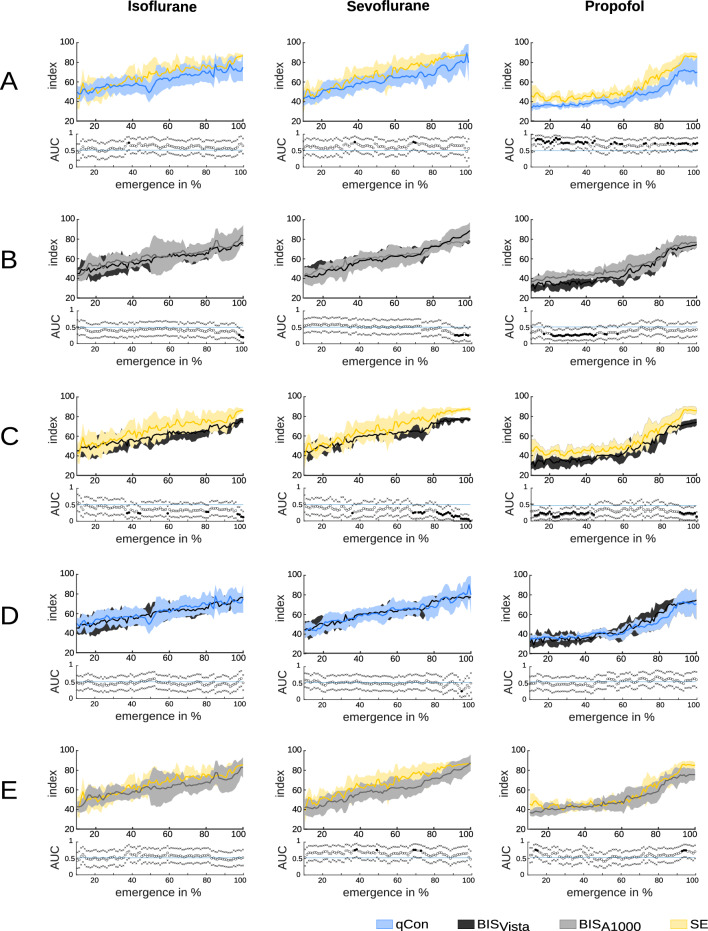
Index values of the different devices **A** SE vs. qCON, **B** BIS Vista vs. BIS A1000, **C**BIS Vista vs. SE, **C** BIS Vista vs qCON, **E** SE vs BIS A1000. Thick line median. AUC plots: A filled circle in black indicates significance and a gray circle indicates a non-significant AUC > 0.7. The non-filled circles indicate AUC < 0.7 with 95 % CIs inclusive 0.5, i.e., there is no effect. The x indicates the upper and lower limits of the 95%CI

When comparing the indices, BIS Vista/A-1000 and qCON showed a clear to high correlation (Spearman Correlation coefficient rs > 0.6) under all regimens. In contrast, SE lacked correlation to the others under propofol anesthesia (rs < 0.6). We also found significant effects between the devices for the following percentages of emergence (Figure 4, Table 2).

**Table 2:** Index values depending on monitoring devices

	Isoflurane (%)	Sevoflurane (%)	Propofol (%)
BIS_Vista_ > qCon	0	1	0
BIS_A1000_ > BIS_Vista_	3	7	29
BIS_A1000_ > qCon	0	0	25
SE > BIS_Vista_	10	25	43
SE > BIS_A1000_	0	6	5
SE > qCon	1	4	51

Percentage of the emergence phase in which one index induces significantly higher values than another one under the respective regimen. The analyzed period is 10–100 % of emergence, as the first 10 % of emergence was cut off due to replaying artifacts

### Observations with indices above 80 or below 60

**Fig. 5:**
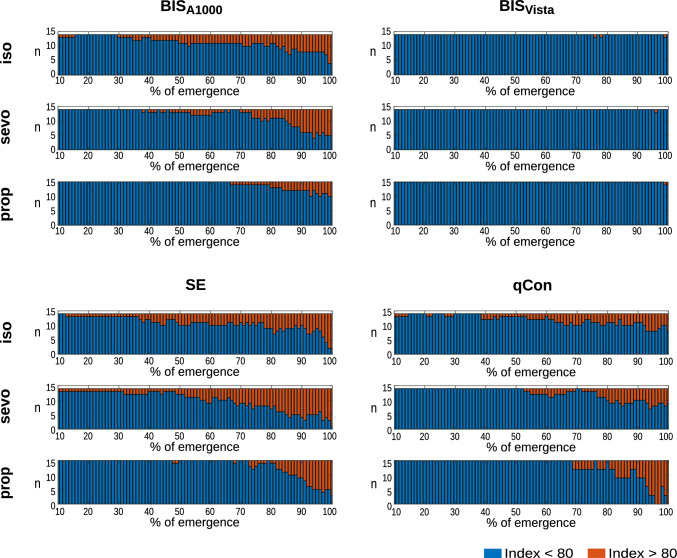
Index > 80 throughout emergence under isoflurane (**A**), sevoflurane (**B**), and propofol (**C**) anesthesia

Index values over 80 indicate a high probability of a conscious patient [28–31]. Excluding the cases where the index did not show values higher than 80 before RoR (52 %), the median time for index > 80 before RoR was 189 s [Max: 1970 s; Min: 10 s] for the sevoflurane regimen, 490 s [Max: 1910 s; Min: 14 s] for the isoflurane regimen, and 105 s [Max: 450 s; Min: 5 s] for TIVA. Isoflurane (p = 0.005) and sevoflurane (p = 0.023), therefore, induced significantly earlier indices > 80 compared to propofol. Under TIVA, indices > 80 are displayed in only 33 % of the patients at RoR. BISVista showed indices > 80 in only eight patients (16 %) overall. This is depicted in Figure 5. Index values between 40 and 60 are considered an adequate range for surgery [28–31]. 7 % of the patients had indices < 60 at the time of RoR. We did observe significant index differences in the last 5 s before RoR between BIS Vista (median: 76) and BIS A-1000 (median: 83) (p = 0.002), BIS Vista and SE (median: 87) (p < 0.001) as well as between qCON (median: 73) and SE (*p* = 0.006). There also were significant differences between the last documented values of the sevoflurane (median: 85) and propofol (median: 76) regimens (p = 0.013).

## Discussion

Our analyses detected substance-specific influences on EEG-based indices during anesthesia emergence, adding information to the already described substance-specific differences in EEG emergence behavior for a limited frequency range [20]. The EEG frequency pattern during anesthesia emergence differed significantly between volatile anesthetics isoflurane and sevoflurane compared to propofol. Volatile anesthetics induced higher power in frequencies above 15 Hz during almost the entire emergence, while propofol caused high power in the delta band. This early activation of the beta oscillations may be caused by cortical activity, which most probably does not imply awareness or even responsiveness [32]. One reason for the substance-specific properties may be the different molecular targets of volatiles and propofol. While propofol predominately acts on the GABA receptor, the spectrum of molecular targets is broader for volatiles [33], causing different spectral EEG patterns during anesthesia maintenance [27] and emergence [20]. These substance-induced differences are also reflected in the index behavior of the monitoring systems. The indices were higher for patients receiving volatiles during the first stages of anesthesia emergence. The BIS is calculated from a proprietary index, but reverse engineering revealed that the information is extracted from the spectrum, focusing on the low gamma band [34]. The state entropy is derived from the spectral entropy [9], and a more uniform power distribution will increase the index. The qCON is derived from combining the power of different frequency bands [8]. The higher beta power may also be one reason the qCON is higher for the volatile groups, as increasing beta leads to an increasing index [8]. In the volatile groups, we also observed index values suggestive of an awake patient significantly earlier than in the propofol group. During anesthesia emergence, EMG may contaminate the EEG. Absent EMG may lead to lower [35] and stronger EMG to higher indices [36]. While the monitoring systems process information up to 47 Hz, like for instance the BIS [7], we limited our spectral analyses to 30 Hz that may contain less EMG information [37], also EMG activity can span the entire frequency range used for EEG analysis [36, 38]. Because the NMB was only used for intubation, the emergence was without NMB. Although, we set a cut-off line at 30 Hz to eliminate the possible impact of muscle activity, the influence can’t be ruled out. It should be investigated in the future regarding the different regimens. Some patients, especially in the BIS and TIVA group, had indices < 60 when responding to verbal commands at the end of emergence. Responsive patients with low indices [39] or EEG features suggesting unconsciousness [40] have been reported. Still, the replay may influence the absolute index values, as the frequency range of the previously recorded and replayed EEG may partially conflict with the range utilized by the algorithms. We rule out a significant influence of blood pressure on the EEG, as intraoperative blood pressure treatment was included in the clinical protocol. Further, the effect of neuromuscular blocking agents [35] can be ruled out, as the time since the last administration before RoR was longer than the duration of the blocking action. Opioids can affect the EEG [41] and the processed indices [42, 43], and since our patients received a combination of anesthetic drugs and opioids, there may have been an impact. This is why we attribute our results not to specific drugs but to the anesthetic regimen. Further studies have to be conducted to specify the findings, using, for example, the same opioid in all groups. Comparing the different monitoring systems among each other, we found a high general correlation, but the comparison with SE led to lower correlation coefficients. This partially contradicts the findings of Schmidt et al. [44], who found a high correlation between the indices but had a different setting and included the perioperative period. We found general differences between the absolute index values over the emergence phase, such as higher SE than BIS and qCON. Although the scaling of the indices is very similar [7–9], they still may behave differently. Numerous publications describe these discrepancies [45, 46]. These results are most likely due to multiple factors, and before the details of the primarily proprietary algorithms are not revealed, we can only speculate about the underlying causes. Our results regarding the dependency of indices on the anesthetic regimen may impact clinical practice. Knowing that volatile anesthetics induce high indices minutes before RoR, the anesthesiologist may reconsider deepening anesthesia again despite indices around or above 80. Additionally, a non-responsive patient under volatile anesthesia with an index already indicating wakefulness should not immediately become a concern, and accelerating the awakening by, e.g., shaking should be avoided as emergence from deep sedation levels is a risk factor for PACU-D and its consequences. Accordingly, a low index during the reduction of propofol should not provide the reassurance of a still deeply sedated patient, as the patient might reach RoR very quickly. Considering the anesthetic regimen and the monitoring system seems essential when using an EEG-based index to monitor the level of anesthesia and make subsequent clinical decisions.

### Limitations

Calculating the indices based on replayed EEG influences of different recording sites can be ruled out, but potential distortions are created as an already processed EEG is inputted. We can report significant differences even for the small homogeneous patient groups, but whether the findings can be transferred to other population groups (elderly, children, obese) has to be investigated. We did not calculate the exact time delay in processing the different devices. Still, assuming a fictive, based on previously published research [11, 13, 14], number of 60 s, distortion of the data cannot be ruled out. In addition, only single-channel frontal EEG was derived, and information about global brain activity is missing, reflecting the clinical practice. Because the study was conducted in a clinical setting, the patients received other medications besides the anesthetic. Hence, the EEG may have been biased to some degree by these other substances. So, our findings strongly point in the direction of substance-specific differences, but (volunteer) studies with only an anesthetic substance being used could be conducted to confirm our results.

### Conclusion

It becomes clear that there are systematic differences regarding the value of an index depending on the drugs and devices used. A specific index value does not always correlate with the same clinical state of consciousness. Thus, to avoid early awakening or anesthesia overdose, when consulting an EEG-based index during emergence, the anesthetic regimen, the monitor, i.e., the index used, and the raw EEG trace should be considered for interpretation before making clinical decisions.

### Supplementary Information

Below is the link to the electronic supplementary material.

Supplementary file1 (DOCX 705 kb)

## Data Availability

Through correspondence with the aouthors.
