# Supplementary material for: Substance-dependent EEG during recovery from anesthesia and optimization of monitoring
Source: J Clin Monit Comput. 2023 Dec 18;38(3):603–12. doi: 10.1007/s10877-023-01103-4 (PMC11164797; doi:10.1007/s10877-023-01103-4)

***Supplemental Information***

| **Patient characteristics** | | | |
| --- | --- | --- | --- |
|  | Propofol (n=15) | Isoflurane (n=14) | Sevoflurane (n=14) |
| **Sex**  w  m | 9 (60%)  6 (40%) | 9 (64%)  5 (36%) | 8 (57%)  6 (43%) |
| **ASA***^a^*  I  II | 10 (67%)  5 (33%) | 10 (71%)  4 (29%) | 7 (50%)  7 (50%) |
| **Weight** [kg] | 70,5 | 90 | 75 |
| **Height** [cm] | 167 | 181,5 | 173 |
| **body mass index** | 25,6 (n=14*^b^*) | 28,1 | 25,9 |
| **Age** | 57 (n=14*^b^*) | 40 (n=13*^c^*) | 41,5 |

**Supplemental Table S1: Patient characteristics.** Median presented for weight, height, BMI, age. *^a^*American Society of Anesthesiologists  *^b^*One patient excluded due to missing data. *^c^*One patient excluded due to unrealistic documented age of 153 years.

**Supplemental Figure 1: Spearman coefficients.** For all patients in total and separated by regimen.


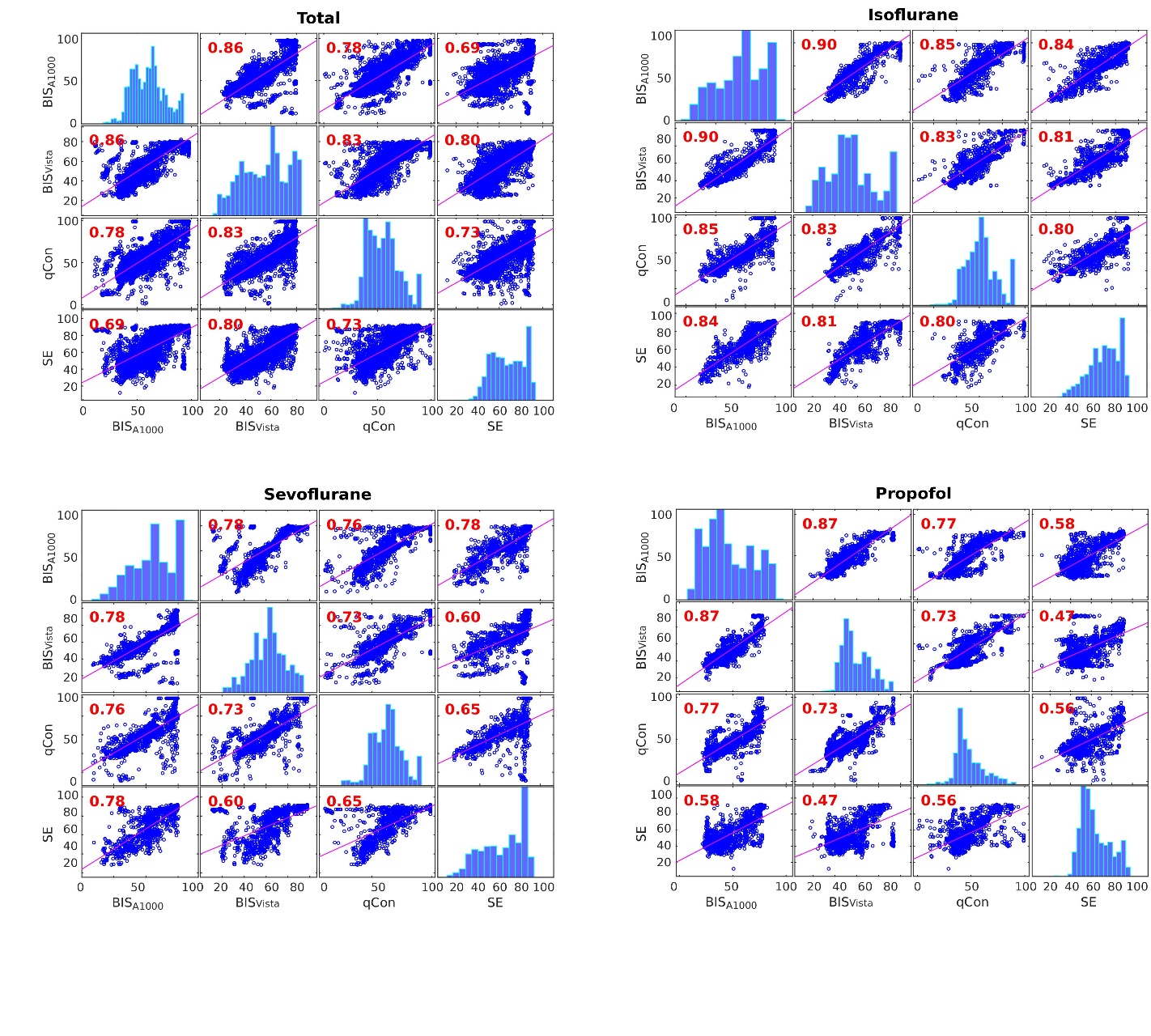

Supplement: Supplementary file 1 — Supplementary file1 (DOCX 705 kb) [file 10877_2023_1103_MOESM1_ESM.docx]
